# Life satisfaction, regret, and death reflection among older adults in Turkey: a qualitative study within Erikson’s framework of ego integrity

**DOI:** 10.3389/fpsyg.2026.1745222

**Published:** 2026-04-15

**Authors:** Ertuğrul Talu

**Affiliations:** Department of Guidance and Psychological Counseling, Ahi Evran University, Kırşehir, Türkiye

**Keywords:** death reflection, Erikson’s ego integrity, life satisfaction, older adults, regret

## Abstract

**Background:**

Erikson’s psychosocial theory of development conceptualizes the final stage of Ego Integrity versus Despair as a crucial process in achieving psychological well-being during late adulthood. However, the cultural expressions of this developmental conflict remain insufficiently explored, particularly in non-Western and collectivist societies such as Turkey.

**Objective:**

This study aimed to examine life satisfaction, regrets, and reflections on death among older adults in Turkey within Erikson’s psychosocial framework of ego integrity.

**Methods:**

Using a qualitative research design, the study analyzed open-ended written narratives collected from 48 participants aged 65 and above, residing in different regions of Turkey. Data were examined through a three-phase thematic coding process. More than 100 unique codes were identified and categorized into twelve overarching themes that represented shared psychosocial experiences.

**Results:**

Participants most frequently expressed gratitude (“I’m glad I did”) for experiences related to family formation, child-rearing, professional achievements, and adherence to moral and religious values. Regrets were largely associated with missed educational opportunities, relational disruptions—particularly within marriage—and health neglect. Reflections on death ranged from faith-based acceptance and existential fear to feelings of incompletion and the desire for a dignified death.

## Introduction

Old age is not merely the final stage of the human life course; rather, it is a multifaceted developmental period in which life experiences are revisited with intensity, the search for meaning deepens, and existential concerns become increasingly salient (Moore and Schow, 2006). Beyond biological aging, this period encompasses profound psychological and social transformations, including the reconstruction of identity, reinterpretation of past experiences, and redefinition of the individual’s roles within the family, society, and culture (Westerhof et al., 2017; Whitbourne et al., 2002). Contemporary gerontological literature suggests that, as individuals grow older, they engage more frequently in reminiscence and life review processes, striving to integrate their personal narratives into a coherent and meaningful whole (Bluck and Alea, 2018; Coleman and O’Hanlon, 2017; Settersten and Mayer, 2020). From this perspective, late adulthood represents a phase of meaning-making that extends well beyond “aging” per se, characterized by the consolidation of one’s life story, alignment with core values, and the negotiation of developmental tasks related to mortality awareness (Hill and Turiano, 2014).

One of the most influential frameworks for explaining psychological development in later life is Erik Erikson’s Psychosocial Theory of Development. The eighth stage of the theory—Ego Integrity versus Despair—represents a critical psychosocial turning point in which individuals strive to evaluate their lives as coherent, worthwhile, and meaningful wholes (Erikson, 1959, 1982). Typically encompassing individuals aged 65 and older, this stage is characterized by the pursuit of life satisfaction, meaning, and inner coherence (Coles, 1970). Erikson (1982) conceptualized this period as the culmination of the developmental process and described it metaphorically as the “harvest time” of all preceding stages (Bauer and McAdams, 2004).

According to Erikson’s lifespan perspective, each developmental stage involves a psychosocial conflict that must be resolved to support healthy personality development. In the final stage of life, this conflict centers on the individual’s ability to accept their life as valuable, coherent, and meaningful, thereby attaining ego integrity—a state of psychosocial maturity (Erikson and Erikson, 1997). Achieving ego integrity entails acknowledging both successes and failures in one’s past, reaching a sense of self-acceptance, cultivating inner peace with past choices, and appraising one’s life narrative as an integrated and meaningful whole (Chang et al., 2008; Van der Kaap-Deeder et al., 2020).

When ego integrity is attained, the virtue Erikson (1963) described as wisdom emerges—a quality reflecting the capacity to accept the finitude of life while maintaining a sense of inner wholeness (Gilleard, 2020). This state of psychosocial maturity has been associated with higher levels of life satisfaction, emotional balance, and psychological well-being in older adults (Ardelt and Jeste, 2022; Etezadi and Pushkar, 2013). Conversely, when this integrative process fails, ego integrity may give way to despair, characterized by regret over the past, perceived missed opportunities, and unresolved disappointments (Lecci et al., 1994; Spirling, 2019). Such experiences can precipitate heightened death anxiety and evolve into a psychological rupture marked by a pessimistic appraisal of life’s meaning (Balasubramanian et al., 2018; Wong, 2000).

Research conducted across diverse cultural contexts provides robust empirical support for Erikson’s conceptualization of ego integrity and despair. In particular, studies from Western Europe, North America, and Asia indicate that individuals with higher levels of ego integrity report greater life satisfaction, enhanced subjective well-being, and stronger acceptance of death (Ryff and Singer, 2013; Van der Kaap-Deeder et al., 2020; Van Hiel and Vansteenkiste, 2009). For example, in Western cultural contexts, individuals tend to evaluate their lives primarily in terms of personal achievements and self-actualization, whereas in collectivist cultures, religious values, family bonds, and social harmony emerge as more salient evaluative criteria (Karasawa et al., 2011; Krause, 2003; Whitbourne et al., 2002). These cross-cultural findings further demonstrate that pathways to ego integrity and the criteria for a “well-lived life” are closely intertwined with culturally embedded value systems (Kagitcibasi, 2017; Markus and Kitayama, 1991).

Within the international literature, retrospective life evaluations—particularly experiences of gratitude and regret—are emphasized as central mechanisms in the development of ego integrity during late adulthood (Rutledge et al., 2024). Gratitude has been identified as a key psychological resource that facilitates positive reappraisal, emotional regulation, and the acceptance of life’s inherent limitations (Emmons and McCullough, 2003). Moreover, cross-cultural research suggests that the resolution of the psychosocial conflict associated with Erikson’s final stage is shaped not only by individual-level factors but also by sociocultural influences such as religious beliefs, familial obligations, and collective memory (Knight and Sayegh, 2010; Krause, 2003).

Research has shown that individuals who achieve ego integrity are able to make sense of life events by integrating both their positive and negative aspects; this also provides the individual with advanced psychosocial maturity indicators such as resilience, generativity, and acceptance of death (Ardelt and Jeste, 2022; McAdams et al., 1997). However, it has been noted that achieving ego integrity is not a universal process; due to social pressures, gender roles, limited opportunities, or generational structural disadvantages, this process can be more challenging in some cultures (Chen et al., 2021; Knight and Sayegh, 2010; Van der Kaap-Deeder et al., 2020).

In culturally hybrid societies such as Türkiye—where traditional, religious, and collectivist values intersect with processes of modernization and individualization—the experience of aging is shaped by the convergence of multiple factors, including gender roles, religious beliefs, and prevailing social norms (Uysal, 2020). Rapid urbanization, economic fluctuations, and transformations in family structures profoundly influence older adults’ life satisfaction, retrospective regrets, and perceptions of death (Özer and Yıldırım, 2024). In particular, retrospective evaluations of family relationships, career decisions, educational opportunities, and health-related choices—often articulated through expressions such as “I am glad I did” or “I wish I had not”—play a decisive role in positioning individuals toward ego integrity or despair (Bilgili and Arpacı, 2014; Kurt et al., 2010). Within this context, family-related achievements are commonly associated with feelings of pride and fulfillment, whereas missed educational or occupational opportunities tend to be linked to regret and self-doubt (Çelik et al., 2018; Kulakçı-Altıntaş and Ayaz-Alkaya, 2020).

An examination of research conducted Türkiye reveals a notable paucity of studies that approach the experience of aging through an individual-centered and culturally contextualized lens. Existing scholarship has primarily focused on quantitative assessments of life satisfaction and well-being, while deeper themes such as older adults’ subjective life narratives, experiences of regret and gratitude, and ways of confronting mortality have received limited scholarly attention. This gap underscores the distinctive value of qualitative research approaches in capturing both the individual and social dimensions of the aging experience.

Against this backdrop, the present study seeks to address a significant gap in the literature by examining older adults’ retrospective self-evaluations of their life experiences and perceptions of death within the Turkish cultural context, grounded in Erikson’s psychosocial framework of ego integrity versus despair. The primary contribution of the study lies in its integration of Erikson’s universal theoretical model with local sociocultural realities, thereby illuminating the dynamic interplay between universal principles of psychosocial development and culturally specific life meanings. In this respect, the study not only probes the cross-cultural applicability of Erikson’s theory but also offers both theoretical and practical insights to inform the development of culturally sensitive social policies and psychosocial support services for older adults.

## Methodology

### Research design

This study employed a qualitative research design to explore how older adults construct meaning from their lives, reflect on their past experiences with satisfaction and regret, and contemplate death. Grounded in an interpretivist paradigm, the research aims to understand individuals’ subjective experiences within their specific sociocultural contexts.

### Participants

The study included 48 participants aged 65 and older, residing in various provinces of Turkey (Kırşehir, Ankara, and Kayseri). Participants were selected through purposive sampling and were required to meet the criteria of being literate, cognitively intact, without any psychiatric diagnosis, and willing to participate voluntarily. The participant profile was diversified to include basic demographic variables such as age, gender, marital status, educational status, socioeconomic status, and cohabiting individuals. In this context, the dataset provides a meaningful framework of the participants’ life experiences. Detailed sociodemographic information about the participants is shown in Table 1.

**Table 1:** Socio-demographic distribution of participants.

Variable	Categories	Frequency (*n*)	Percentage (%)
Gender	Female	23	47.9
	Male	25	52.1
Marital status	Married	31	64.6
	Widowed	15	31.3
	Other	2	4.2
Educational level	Secondary school or below	36	75.0
	High school or above	12	25.0
Economic status	Low	7	14.6
	Middle	35	72.9
	High	6	12.5
Living arrangement	With spouse in their own home	29	60.4
	Alone	9	18.8
	With children	10	20.8
Age	Minimum–maximum	65–82	—
	Mean ± SD	71.1 ± 4.22	—

Of the 48 participants, 47.9% were female (n = 23) and 52.1% were male (n = 25), indicating a balanced gender distribution. In terms of marital status, the majority were married (64.6%), followed by widowed (31.3%) and other categories (4.2%). Regarding educational attainment, 75% of participants had completed middle school or less, while only 25% had completed high school or higher education. This distribution suggests that the sample generally consisted of individuals with low educational backgrounds.

When asked about their perceived economic status, 14.6% described their financial situation as “low,” 72.9% as “moderate,” and 12.5% as “high.” These findings indicate that most participants identified as belonging to the middle-income group. Concerning living arrangements, 60.4% reported living with their spouse in their own home, 18.8% were living alone, and 20.8% were living with their children. These responses suggest that the majority of participants were part of a nuclear family household structure. The participants’ ages ranged from 65 to 82, with a mean age of 71.1 years (SD = 4.22), confirming that the sample consisted of older adults.

### Data collection procedure

Data were collected between March and April 2025 through face-to-face interviews using a semi-structured questionnaire developed by the researcher. At the beginning of the interview, participants were asked if they wanted to read and write answers to the questions on the form themselves; accordingly, 26 participants requested reading and writing assistance from the researcher, while 22 participants did this themselves. The form consisted of three open-ended items based on the core psychosocial themes of Erikson’s “Ego Integrity versus Despair” stage:

1. “What are the experiences or life events that you feel proud of — the things you are glad to have done or lived through?
2. “What are the experiences or events in your life that you regret — things you wish you had not done or that had not happened?”
3. “How do you think and feel about death and the meaning it holds for you?”

In addition to the open-ended items, the form included several closed-ended questions to collect basic demographic information such as age, gender, marital status, educational background, economic status, and current living arrangements. Male participants were mostly recruited from city parks, retirement coffeehouse, and mosque courtyards, while female participants were reached primarily through home visits. To prevent married couples from influencing each other during the response process, only one of them (on a voluntary basis) was included in the study. Each interview lasted approximately 20 to 30 min. No financial compensation was provided for participation. Throughout the data collection process, participant responses were analyzed thematically, and interviews were terminated once data saturation was reached, meaning no new themes or units of meaning emerged. This approach is used as a fundamental criterion for determining sample size in qualitative research (Guest et al., 2006).

### Ethical considerations

The study was conducted in line with the Declaration of Helsinki, with design and methodology reviewed and approved by the ethics committee of the author’s university. All participants signed informed consent forms voluntarily prior to the study. The researcher explained the study’s purpose and importance and assured participants of confidentiality and privacy protection.

### Data analysis

The data were analyzed using Braun and Clarke’s (2006) six-phase thematic analysis framework. Coding was conducted line by line based on participants’ written responses. The analysis began with explicit semantic codes representing clearly articulated meaning units and gradually progressed toward deeper, more latent structures. While the overall approach to coding was largely inductive, the final organization of themes was informed by the theoretical framework of Erikson’s “Ego Integrity versus Despair” stage.

The coding was conducted in 3 cycles:

1. Descriptive Coding: Identification of direct verbal expressions.
2. Semantic and Interpretive Coding: Identification of expressed values, emotional tones, and identity markers.
3. Pattern Coding: Grouping individual codes into meaningful clusters and structuring themes.

As a result of this process, themes were created for each question that were distinct within themselves but related to each other. The themes constitute psychosocial structures that reflect not only superficial concept clusters but also fundamental areas of meaning related to the life cycle of older adults.

Throughout the analysis, attention was paid to the sociocultural context in which the narratives were situated. Interpretations were carried out with methodological rigor, theoretical sensitivity, and a commitment to reflexivity to minimize researcher bias. The coding and theme development processes were iteratively reviewed to ensure coherence and thematic integrity. All codes and their associated subcodes were systematically organized in Microsoft Excel to maintain transparency and traceability of the thematic analysis. This structured documentation served as a foundational tool for analytical consistency. To visualize the relationships between major themes and their subcodes, the Python programming language was employed, utilizing the libraries pandas, networkx, matplotlib, and collections. For each overarching theme, a multidimensional network graph was generated to illustrate its underlying subcodes.

### Coding reliability

The coding process was independently conducted not only by the primary researcher but also by an external expert who provided methodological support to the study. The two sets of codes were compared and cross-checked; any discrepancies were discussed until consensus was reached, ensuring internal consistency and reliability.

The credibility of the study was reinforced through a transparent analytical process, the verification of code–theme relationships, and the researcher’s methodological diligence. All coding decisions were grounded in traceable, explanatory data trails, and analytical interpretations were directly supported by participants’ verbatim expressions. To enhance data richness and trustworthiness, each theme was constructed using data derived from multiple participants, thereby ensuring source triangulation.

## Results

The analysis yielded a total of 12 main themes across the three guiding questions. Each theme was constructed through the clustering of semantically related content. Table 2 presents the themes corresponding to each question along with illustrative participant statements.

**Table 2:** Themes and ıllustrative content derived from participants’ responses.

GuidingQuestion	Theme	Content summary
Question 1 – sources of pride in life	Family and intergenerational meaning	Marriage, having children, choosing the rights pouse, establishing strong familyties, supporting children’s education, raising children, contributing to Grand children’s lives, maintaining familial bonds.
	Occupational and economic independence	Having a profession, retirement, securing employment, achieving financialin dependence, working as a teacher, making a difference in students’ lives, contributing to others.
	Pride in lifestyle choices	Making one’s own life decisions, avoiding harmful habits, gaining individual experiences such as traveling abroad.
	Moral and spiritual fulfillment	Finding meaning through morally and spiritually grounded actions such as being just, showing compassion, helping others, engaging in religious practices.
Question 2 – life regrets	Missed life opportunities	Regrets over lost educational opportunities, poor time management, careeror financial decisions, and missed chances that’s haped life direction (e.g., migration).
	Relational and familial disruptions	Family conflicts, regret overs pouse choice, emotional distance from children, deterioration of social bonds.
	Health and bodily regrets	Regret over unhealthy habits, neglect of physical well-being, past decisions that negatively affecte done’she alth.
Question 3 – thoughts on death	Attachment to life and a sense of incompletion	Persistent. Desires and expectations despite nearing death; statements like “I have not seen my Grand children yet,” or “There are stil things I want to experience.”
	Fears and perceived uncertainty surrounding death	Anxiety about the unknown aspects of death, including its timing, manner, and the fear of dying alone; e.g., “I do not want to die,” “Every one will forget me,” “Uncertainty frightens me.”
	Faith-based acceptance and surrender	Perceiving death as God’s will, a part of destiny, or the beginning of the after life journey; e.g., “Everything is from God,” “I want to die with faith,” “Death is a transition.”
	Peacefulness and readiness for death	Acceptance of death as a calm, inevitable, and natural process; expressions like “I’m ready,” “My time has come,” “Death is part of nature’sorder.”
	Wishes and expectations about the manner of death	Desiresfor a painless, dignified death without dependency on others; e.g., “I do not want to suffer,” “I want to die without being a burden.”

Responses to all three open-ended questions were carefully examined line by line and segmented into meaningful units. From these units, open codes were generated based on the emotional, behavioral, and value-laden content present in participants’ narratives. These codes were predominantly inductive in nature, derived directly from participants’ expressions and constructed using a bottom-up approach. For instance, the response “I raised my children and helped them build careers” gave rise to multiple codes, including “parental pride” and “contribution to education.”

Over 100 distinct codes were identified and subsequently grouped according to semantic proximity and content similarity. The main principles guiding the clustering process were as follows:

Content-level consistency: Codes reflecting similar meanings were grouped together. For example, “having children,” “motherhood,” and “raising children” were classified under the same thematic category.

Structural relationships: Certain codes did not directly describe observable events but rather reflected the emotional or symbolic dimensions associated with those events. These were linked to higher-order thematic structures centered on meaning. For example, the expression “I worked hard and I’m glad I was able to provide well for my children” was associated with both “economic success” and “familial fulfillment,” but was ultimately subsumed under the broader theme of “Life Meaning Through Family.”

Themes were constructed not only on the basis of content similarity but also with reference to theoretical coherence. During this process, thematic categories were refinedeither expanded or narrowedand overlapping or redundant themes were merged. For example, initially distinct themes such as “religious submission,” “readiness,” and “acceptance” were integrated under the more comprehensive label of “Faith-Based Acceptance and Surrender.” Similarly, codes such as “having children,” “motherhood,” and “raising offspring” were unified within the overarching theme of “Family and Intergenerational Meaning.”

### Question 1: life events that elicit pride (“I’m glad I did/experienced it”)

Participants’ responses to the question “What are you proud ofthings you are glad tohave done or experienced?” provide rich insight into how older adults construct meaning in retrospective self-evaluations, which life experiences they deem meaningful, and how they articulate life satisfaction through specific practices. The narratives of all 48 participants were thematically analyzed and categorized under four primary themes: Family and Intergenerational Meaning, Occupational Identity and Social Contribution, Individual Lifestyle and Values, and Moral and Spiritual Fulfillment.

### Theme 1: family and intergenerational meaning

The vast majority of participants described family formation, having children, and raising them to become contributing members of society as the most meaningful aspects of their lives. A total of 26 participants explicitly expressed views related to this theme, underscoring the centrality of familial bonds in their life evaluations. These narratives not only emphasize the presence of a family structure but also highlight its qualitative aspectsemotional bonding, shared growth, sustained effort, childrearing, and marital continuity. Participants interpreted these experiences not merely as personal accomplishments but as the fulfillment of social responsibilities, demonstrations of moral adequacy, and acts of intergenerational value transmission.

P29/F: “*Raising my children to be well-mannered and moral individuals.”*

P20/F: “*Becoming a mother and raising my children myself—they are my greatest source of pride.”*

For some, these experiences transcended personal satisfaction and were seen as the successful completion of a “life mission.” The spiritual fulfillment derived from parenthood was conveyed with deep emotional tone and was often framed as the meaningful return on past sacrifices, contributing to their current sense of peace.

P26/M: “*I raised my children and brought them to a good place in life.”*

P42/F: *“I worked for years to provide a good life for my children.”*

Marriage, likewise, was not only described as a romantic partnership but as a relationship rooted in solidarity, shared struggle, and collective action for the sake of the children’s future. Many participants expressed gratitude and pride toward their spouses, and marriage was often discussed alongside parenting as part of a unified life structure that served as a primary source of identity and belonging.

P33/F: “*Marrying my spouse. Building a home together*.”

P14/F: “*I’ve never been estranged from my children. I’m glad we became such a family.”*

**Figure 1:**
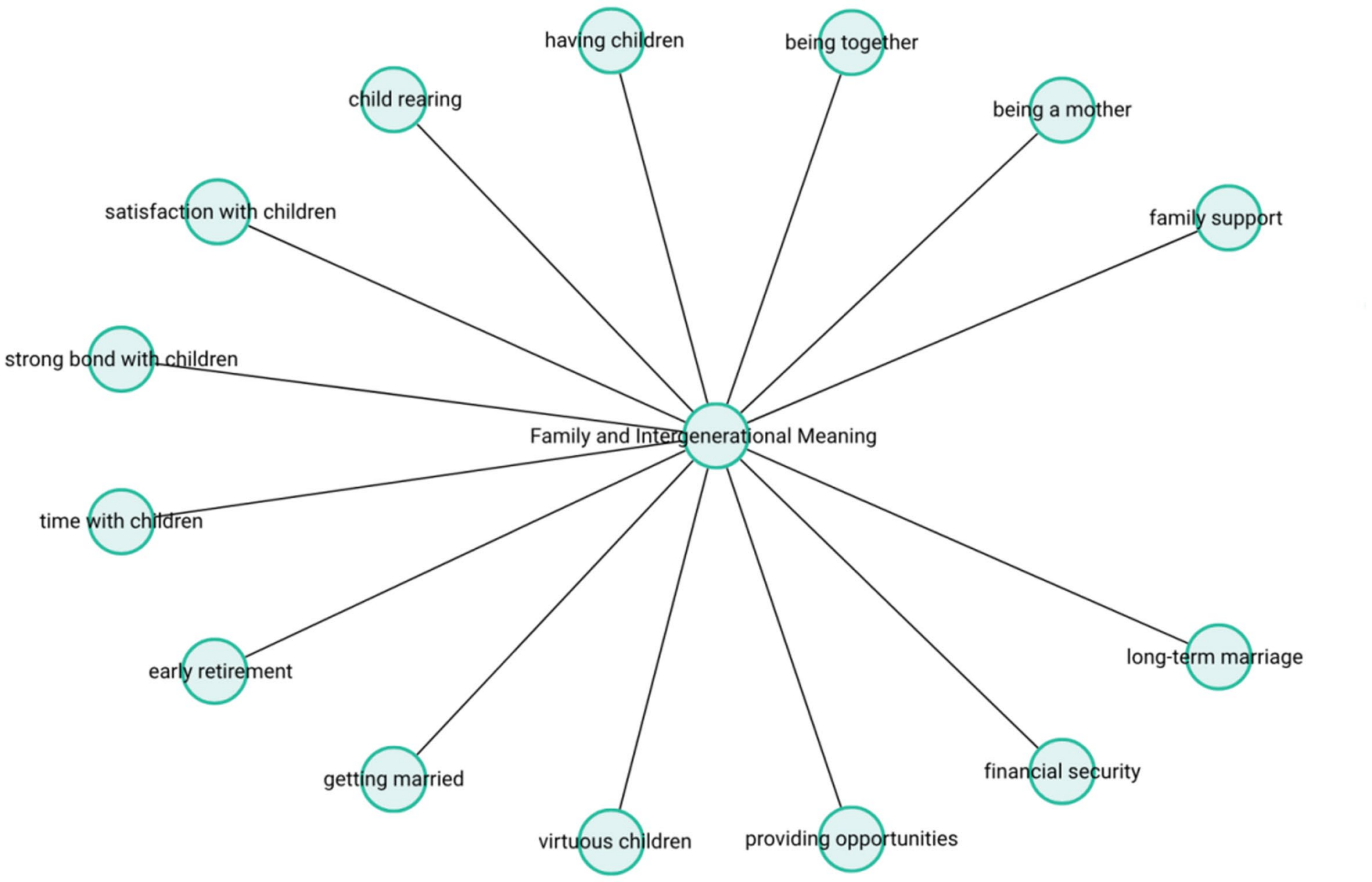
Family and inter generational meaning.

These findings echo Erikson’s conceptualization of the Ego Integrity versus Despair stage, in which the search for meaning in old age often revolves around familial accomplishments, intergenerational continuity, and the internalization of socially sanctioned values. Especially among female participants, the role of motherhood emerged not only as a cornerstone of personal identity but also as the most meaningful expression of their social role. Overall, this theme illustrates the pivotal role that intra-family dynamics play in shaping life meaning, and how satisfaction in later life is deeply informed by the experience of continuity across generations. The theme-subcode network (codemaps) of “Family and Intergenerational Meaning” is presented in Figure 1.

### Theme 2: occupational and economic Independence

A significant number of participants identified having a profession, achieving financial independence, and contributing to their family and community as central elements that gave meaning to their lives. In total, 13 participants described their occupational experiences not merely as a means of livelihood, but as a foundational component of both personal identity and social responsibility. Some participants emphasized the self-respect gained through formal education and professional training, while others highlighted the satisfaction of building a life through hard work and perseverance:

P8/M: “*I acquired a profession and became someone beneficial to my country*.”

P36/M: “*Having state-sponsored insurance and securing my financial stability puts my mind at ease.*”

For some, their career was not only a personal accomplishment but also a set of values passed down across generations. Long-standing professional engagement was often narrated with a sense of belonging, social recognition, and a retrospective feeling of having “achieved something meaningful” in life:

P6/M: “*Choosing the profession of architecture and continuing the cultural tradition of yarencilik in Simav for 40 years… That’s my real source of pride.”*

References to “working hard” and “being part of something” appeared frequently in participants’ life stories, symbolizing not only personal fulfillment but also the desire to secure the well-being of their children. These expressions suggest that occupational identity functioned not only as a career path but also as a critical platform for fulfilling familial obligations, contributing to society, and cultivating self-respect:

P46/M. *“I worked hard and earned money.”*

P5/M: “*I provided a good life for my children and my wife financially.”*

**Figure 2:**
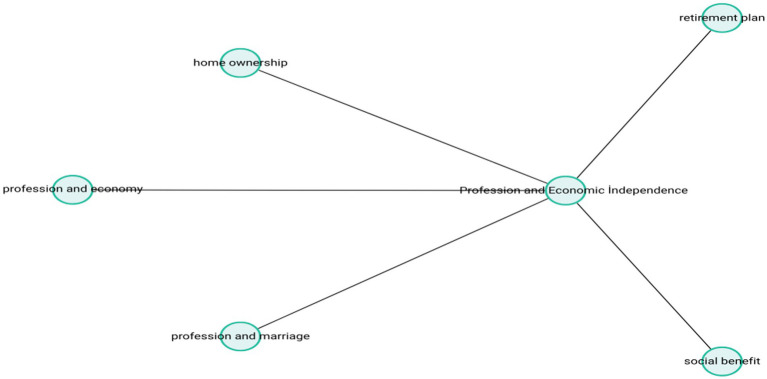
Profession and economic independence.

The narratives under this theme closely reflect Erikson’s psychosocial concept of generativitythe need to be productive and to contribute meaningfully to others. Through their professions, participants appeared to have developed not only a strong personal identity but also a sense of being socially valued individuals. The theme-subcode network (codemaps) of “Profession and Economic İndependence” is presented in Figure 2.

### Theme 3: pride in lifestyle choices

This theme reflects participants’ appreciation of their unique life choices, satisfaction with their lifestyle, and commitment to personal values. A total of four participants expressed views aligned with this theme. Their responses emphasized a sense of contentment derived from making autonomous decisions, living a simple life, and arriving at old age without significant regret:

P31/M: “*I’m glad I made my own choices, just as I wanted.”*

P12/M: *“I’m glad I left the village and moved to the city.”*

For some, the meaning of life was situated in personally meaningful domains such as the simplicity of rural life, international experiences, or enduring friendships:

P24/F: “*I’m glad I had good days in my village.*

P45/M: “*I’m glad I went to France.”*

Personal discipline and a strong sense of individual responsibility also emerged as key sources of pride for some participants. These narratives highlight how value-based decisions and self-directed lifestyles contributed to a subjective sense of life satisfaction and meaning.

**Figure 3:**
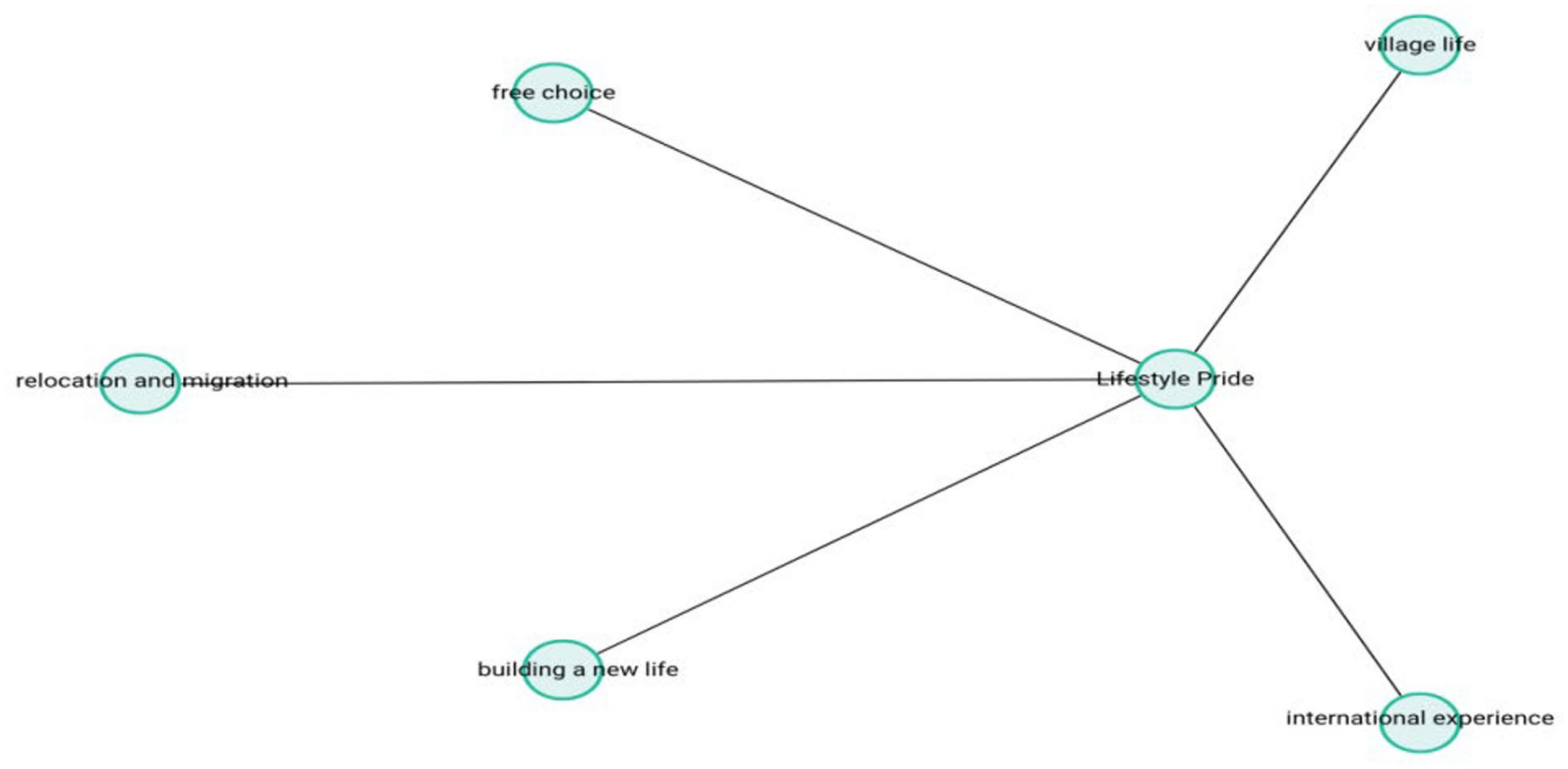
Lifestyle pride.

The theme-subcode network (codemaps) of “Lifestyle Pride” is presented in Figure 3.

### Theme 4: moral and spiritual fulfillment

This theme illustrates how participants derived meaning from their adherence to moral principles, sense of responsibility toward others, and religious devotion. 5 participants offered responses that fell within this thematic category. Several participants emphasized virtues such as justice and compassion as central to their lives and identified them as personal sources of pride:

P10/M: “*I have always acted justly and shown compassion. That’s why I’m proud of myself.”*

For others, caregiving roles within the family especially acts of loyalty and support toward aging parents were described as deeply meaningful:

P21/F: “*I’m proud that I stayed by my sick mother’s side and took care of her.”*

P22/M: “*I’m glad I paid off my sick father’s debts and made him happy*.”

Religious practices and faith-based commitments were also cited as significant contributors to life satisfaction. These participants viewed continued engagement in worship and religious education as meaningful on both spiritual and moral levels:

P43/M: “I’m glad I attended Qur’an school.”

P30/M: “I helped people and did good deeds.”

**Figure 4:**
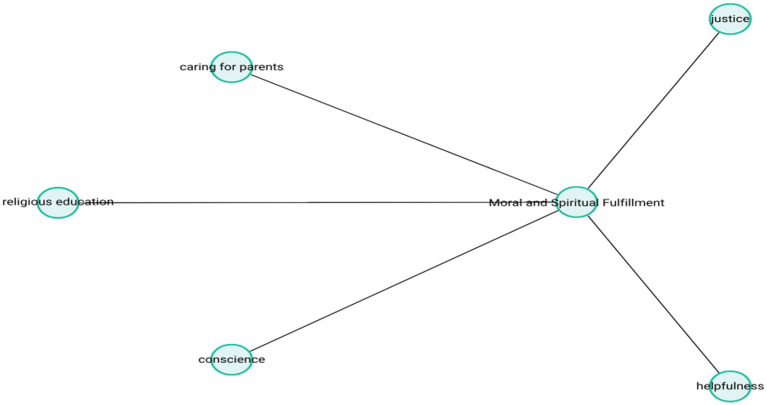
Moral and spiritual fulfillment.

Taken together, these accounts suggest that participants viewed their lives not only as personally fulfilling but also as morally successful. Helping others, fulfilling familial responsibilities, and remaining true to one’s faith emerged as foundational elements of a meaningful life. The theme-subcode network (codemaps) of “Moral and Spiritual Fulfillment” is presented in Figure 4.

### Question 2: life regrets (“I wish I had not done/experienced that”)

Participants’ retrospective reflections, often framed through the lens of “I wish,” pointed toward a wide range of internal reckonings. These reflections were categorized under three primary themes: Missed Life Opportunities, Relational and Familial Disruptions, and Health and Bodily Regrets. Collectively, these themes offer rich insight into how older adults evaluate their past decisions, confront lost opportunities, process interpersonal tensions, and make sense of their physical well-being over the life course.

### Theme 1: missed life opportunities

More than half of the participants described their deepest regrets as stemming from missed opportunities in education, early marriage, poorly managed financial resources, or inadequate time management. A total of 31 participants articulated views aligned with this theme. These expressions reflect a growing awareness that they may have by passed experiences that could have contributed to a “better life.” Many participants emphasized the importance of education, expressing that familial pressures or early marriage prevented them from pursuing further schooling:

P36/M: *“I wish I had been able to continue school. I never wanted to drop out.”*

P34/F: *“I wish I had not gotten married at an early age and continued my education.”*

Similarly, there are participants who say they missed the opportunity to secure their economic future.

P13/F: *“I wish I had saved money back then.”*

P18/M: *“I should have made better use of my financial resources.”*

Some participants, only later in life, recognized the value of time and regretted not dedicating more of it to themselves or personal growth:

P42/F: *“I wish I had taken more time for myself while working so much.”*

P31/M: *“I should have used my time betterlike pursuing education.”*

**Figure 5:**
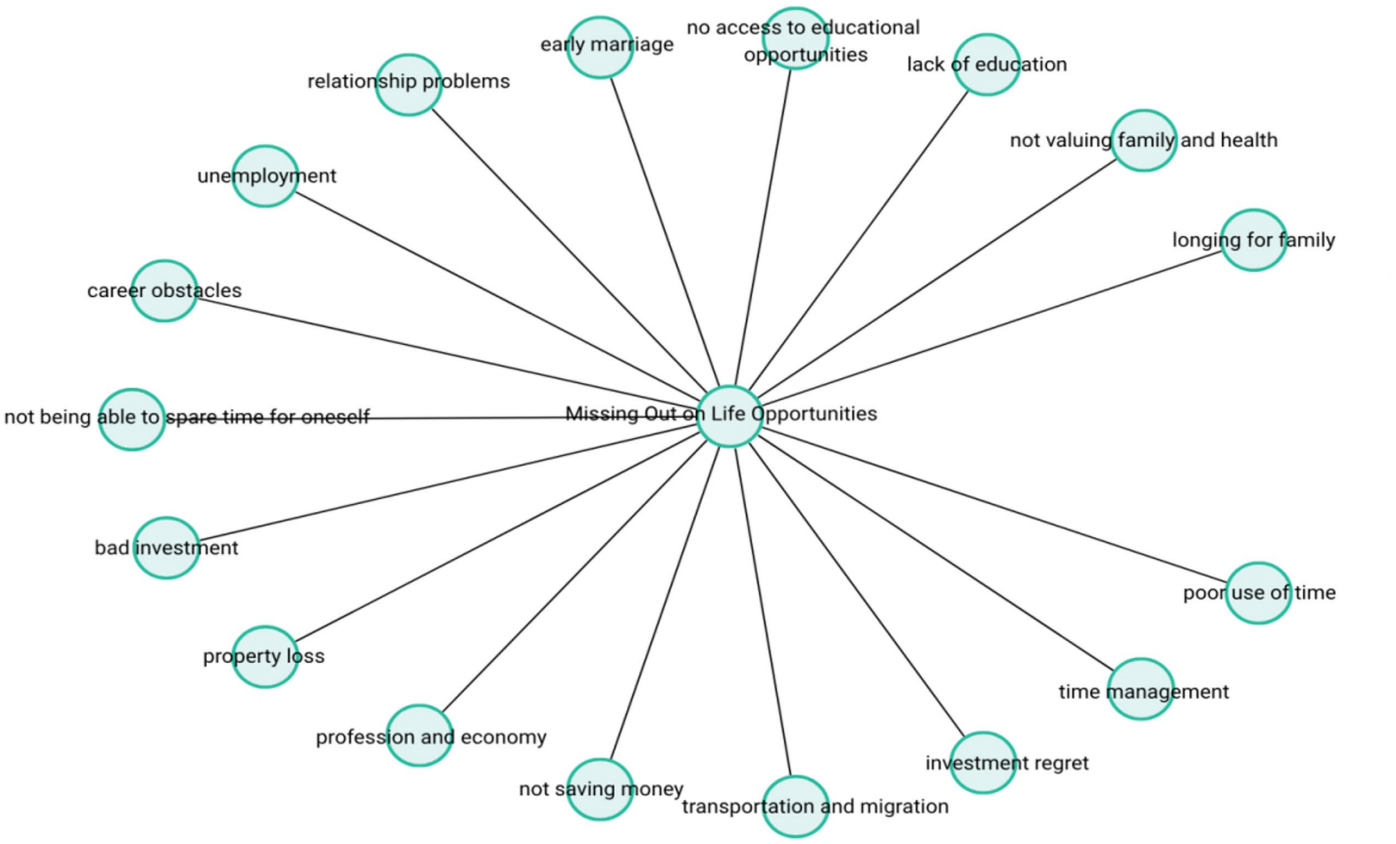
Missing out on life opportunities.

These narratives suggest that participants were aware of how their past choices contributed to current limitations, and this awareness had evolved into a form of internal self-inquiry and existential reflection. The theme-subcode network (codemaps) of “Missing Out on Life Opportunities” is presented in Figure 5.

### Theme 2: relational and familial ruptures

This theme encompasses participants’ disappointments related to marriage, strained emotional ties with spouses or children, and broader familial conflicts. 11 participants reported views associated with this category. Some participants indicated they were forced into unwanted marriages, which negatively affected their lives:

P11/F. *“Being married to someone I did not want.”*

P14/F. *“I wish I had not married my spouse.”*

Beyond marital relationships, communication breakdowns and emotional distance within the family also emerged prominently:

P19/F: “*Being too kind to people.”*

P5/M: *“I wish I had maintained a closer relationship with my children.”*

Some participants deeply regretted not spending more time with deceased family members, such as siblings or parents:

P3/M: *“I wish I had spent more time with my brother before he passed away.”*

**Figure 6:**
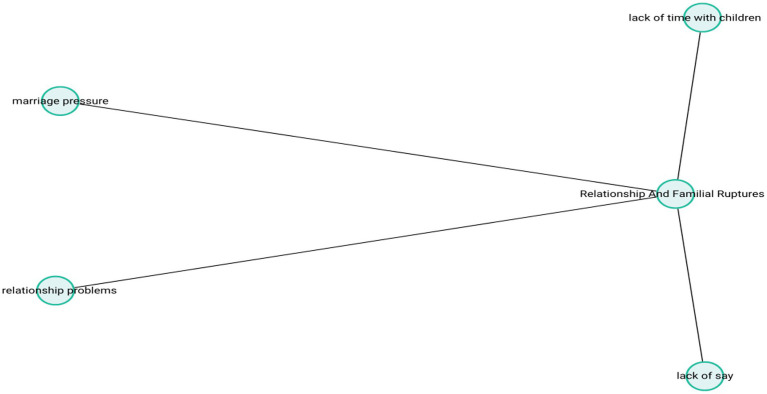
Relationship and familial ruptures.

These expressions reflect late-life introspection regarding the quality of interpersonal relationships and unresolved emotional gaps that can no longer be repaired, often accompanied by a sense of quiet sorrow or guilt. The theme-subcode network (codemaps) of “Relationship and Familial Ruptures” is presented in Figure 6.

### Theme 3: health and bodily regrets

This theme highlights regret over neglecting health, developing harmful habits (particularly smoking and alcohol use), and failing to maintain proper physical care. 6 participants explicitly reflected on these issues, and although fewer in number, the depth and emotional tone of their narratives made this theme particularly salient.

P30/M: “*I wish I did not smoke or drink alcohol. —my lungs are ruined.”*

P1/F: *“I wish I had had surgery on my leg in previous years.”*

Some participants linked their declining health in later life to earlier neglect or fate, framing their experiences with statements such as “I always neglected myself,” which reflect both physical and emotional dimensions of self-care.

P12/M: *“I wish I had cared about my health and gone to the doctor on time.”*

P29/F. “*If only I had not neglected myself, I could have been healthier. But this was also my destiny.”*

**Figure 7:**
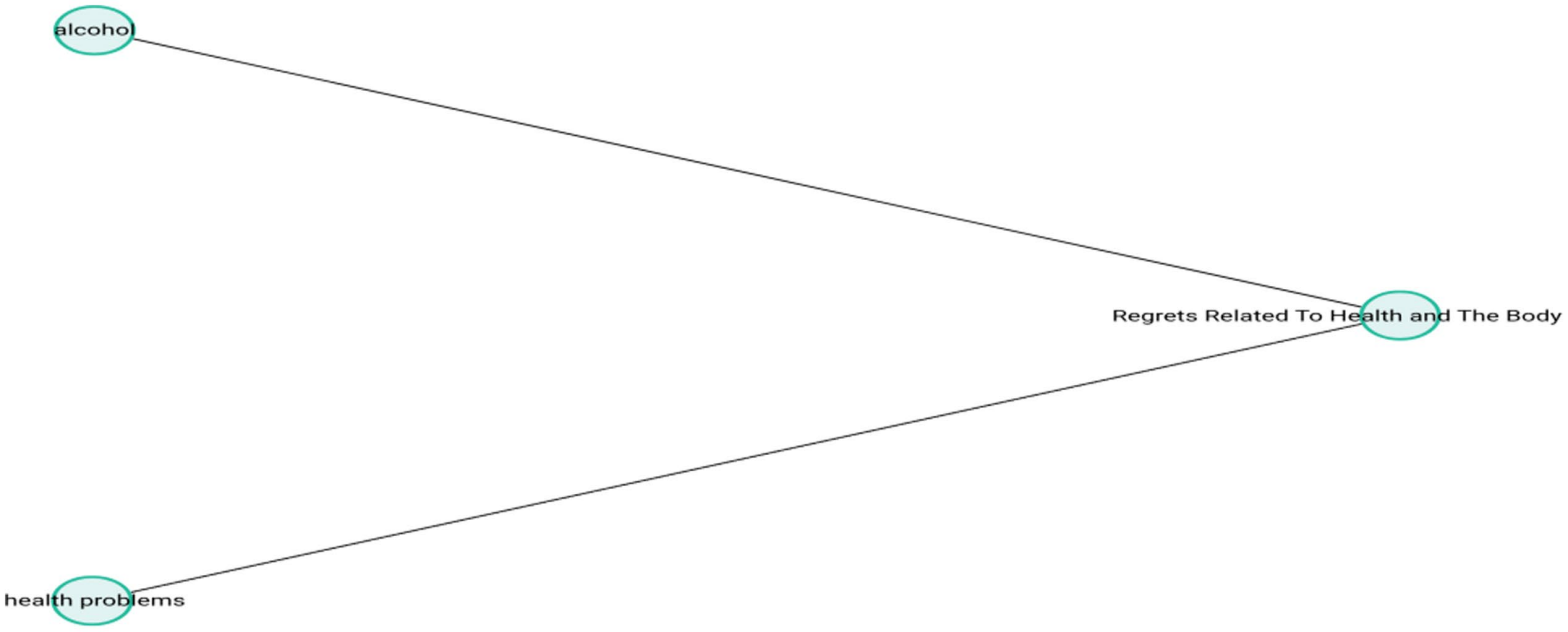
Healthy and body regrets.

The theme-subcode network (codemaps) of “Health and Body Regrets” is presented in Figure 7.

### Question 3: thoughts on death

This section sheds light on how older adults conceptualize death during late adulthood, along with the emotional, cognitive, and faith-based dimensions that accompany this process. Thematic analysis revealed five overarching themes.

### Theme 1: faith-based acceptance and surrender

Many participants viewed death as part of a divine and inevitable order and expressed their acceptance of this reality through a religious lens. A total of 13 participants articulated perspectives consistent with this theme. Their responses emphasized that death is ordained by God, should not be feared, and that what matters most is to die with faith and spiritual readiness.

P33/F: *“It is God’s command—every living being will taste death*.”

P3/M: *“I want to die as a believer, when the time comes, and I hope it will be an easy process.”*

P44/M: “*It’s God’s will. I’m ready.”*

A salient subtheme was the belief that death is not the end of life, but rather the beginning of an afterlife. In this view, death is understood as a meaningful transition rather than a final cessation:

P48/F: *“It’s the moment I will reunite with my father.”*

**Figure 8:**
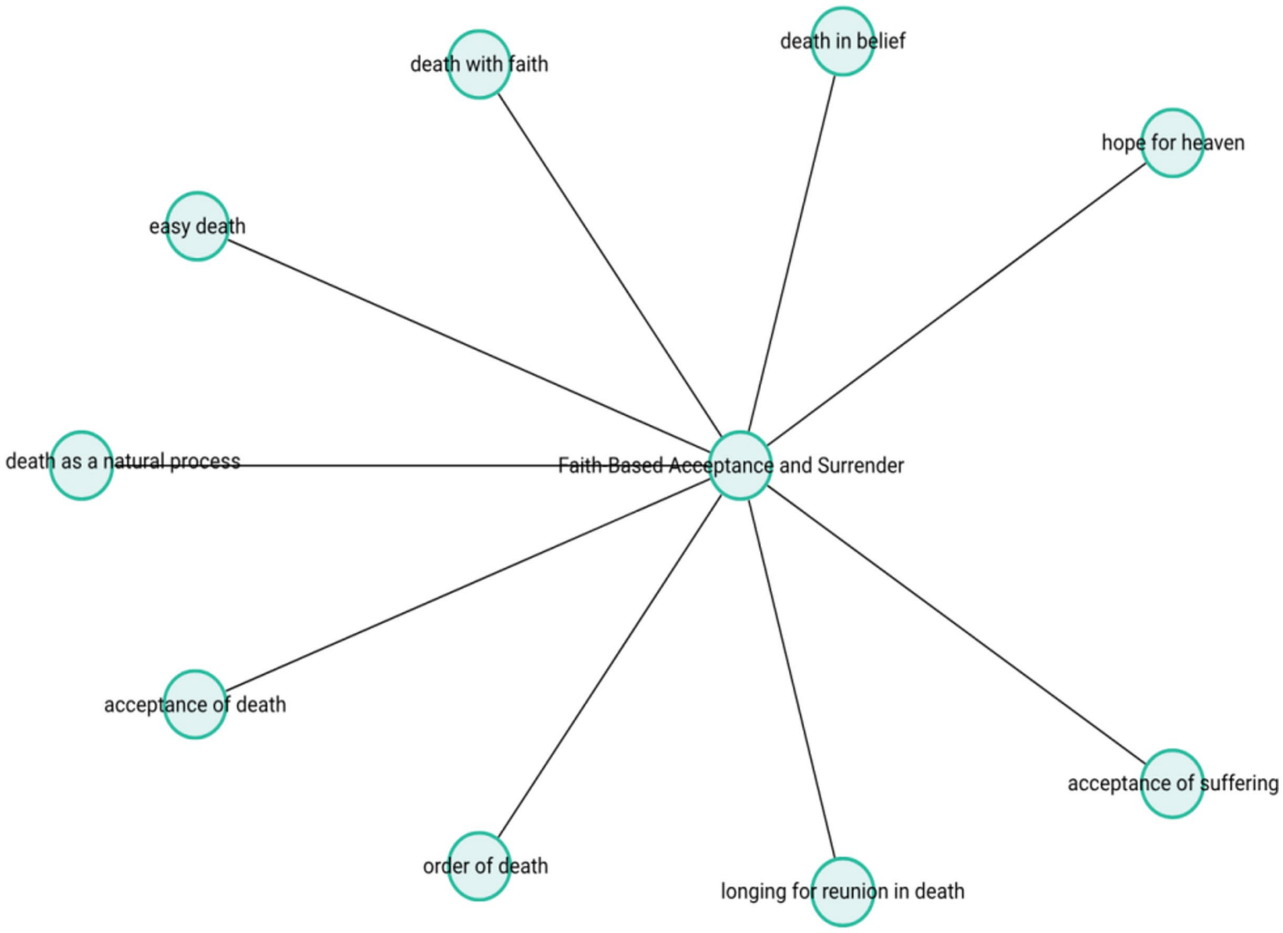
Faith-based acceptance and surrender.

The theme-subcode network (codemaps) of “Faith-Based Acceptance and Surrender” is presented in Figure 8.

### Theme 2: wishes and expectations regarding the manner of death

This theme reflects participants’ desires concerning how and when death might occur. A total of 12 participants expressed views aligned with this category. The notion of a “good death” emerged as centralcharacterized by being painless, maintaining dignity, and not becoming a burden on others:

P27/F: *“A good death, without becoming dependent on others.”*

P37/F: *“To die without needing help from anyone.”*

P8/M: *“I want a painless death.”*

P40/M: *“To live healthily and have a peaceful end.”*

**Figure 9:**
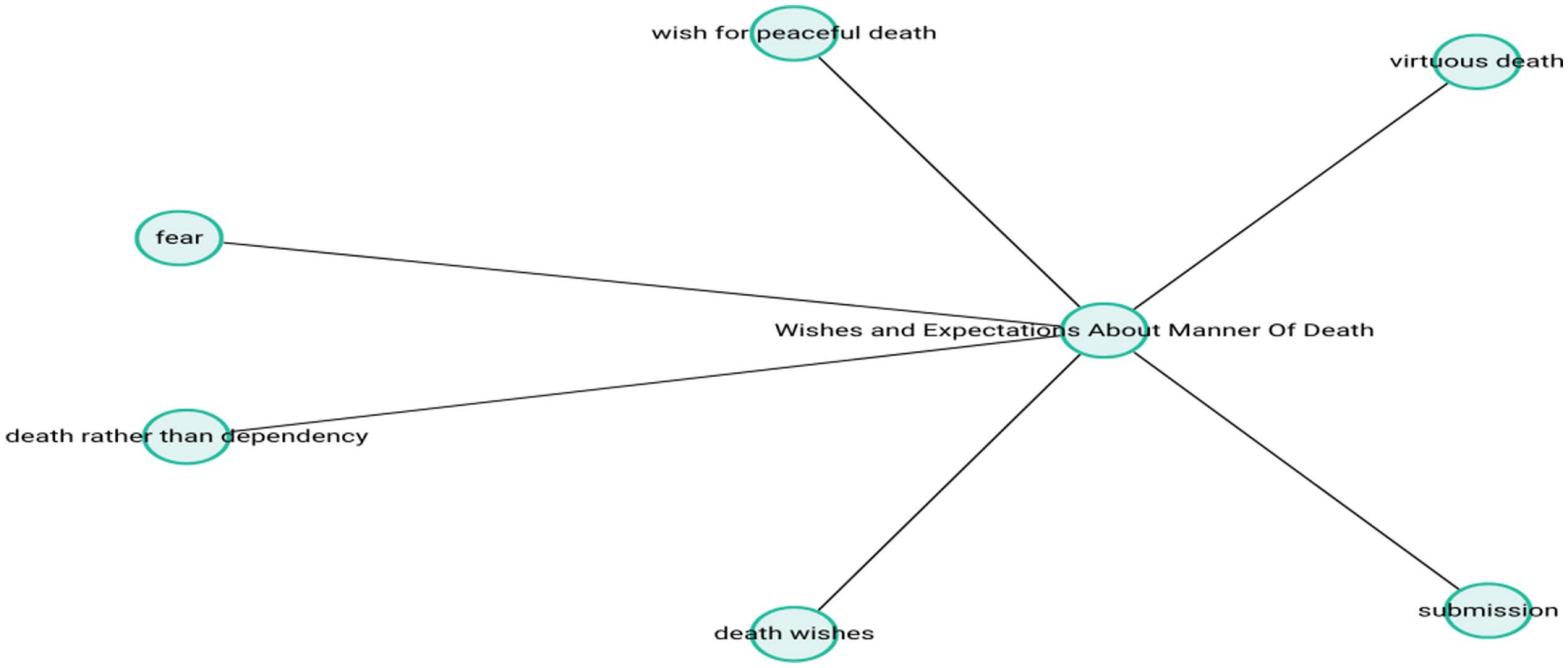
Wishes and expectations about manner of death.

These accounts reflect a strong desire to preserve both physical and psychological integrity and to experience death as a dignified process that minimizes trauma for loved ones. The theme-subcode network (codemaps) of “Wishes and Expectations About Manner of Death” is presented in Figure 9.

### Theme 3: peacefulness and readiness for death

Some participants reported achieving emotional reconciliation with the reality of death and accepting it as a natural part of life. A total of 7 participants expressed such perspectives, often portraying death as a logical and expected endpoint to a completed life cycle:

P10/M: *“Death is a law of nature. It is the expected end for all beings—everything born must die, everything new will grow old.”*

P22/M: “*When my time comes, I will die. I’m not afraid.”*

P31/F: *“Life certainly has an end; humans will die one day. I’m ready.”*

**Figure 10:**
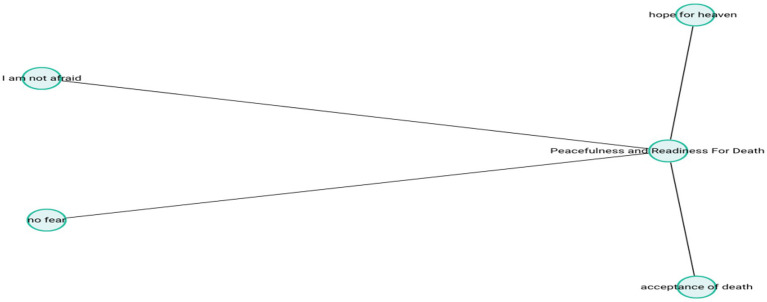
Peacefulness and readiness for death.

These reflections suggest that participants have developed cognitive maturity around the concept of death, accompanied by emotional equilibrium. The theme-subcode network (codemaps) of “Peacefulnessand Readiness forDeath” is presented in Figure 10.

### Theme 4: fears and perceived of uncertainty about death

Some participants described death as frightening, uncertain, and something to be avoided. A total of 8 participants contributed to this theme. Common concerns included the fear of dying, uncertainty about how it will happen, and anxiety about leaving loved ones behind:

P18/M: *“Death scares me.”*

P29/F: *“I do not want to upset those left behind.”*

P2/F: *“It fills me with fear. Everyone will forget me.”*

**Figure 11:**
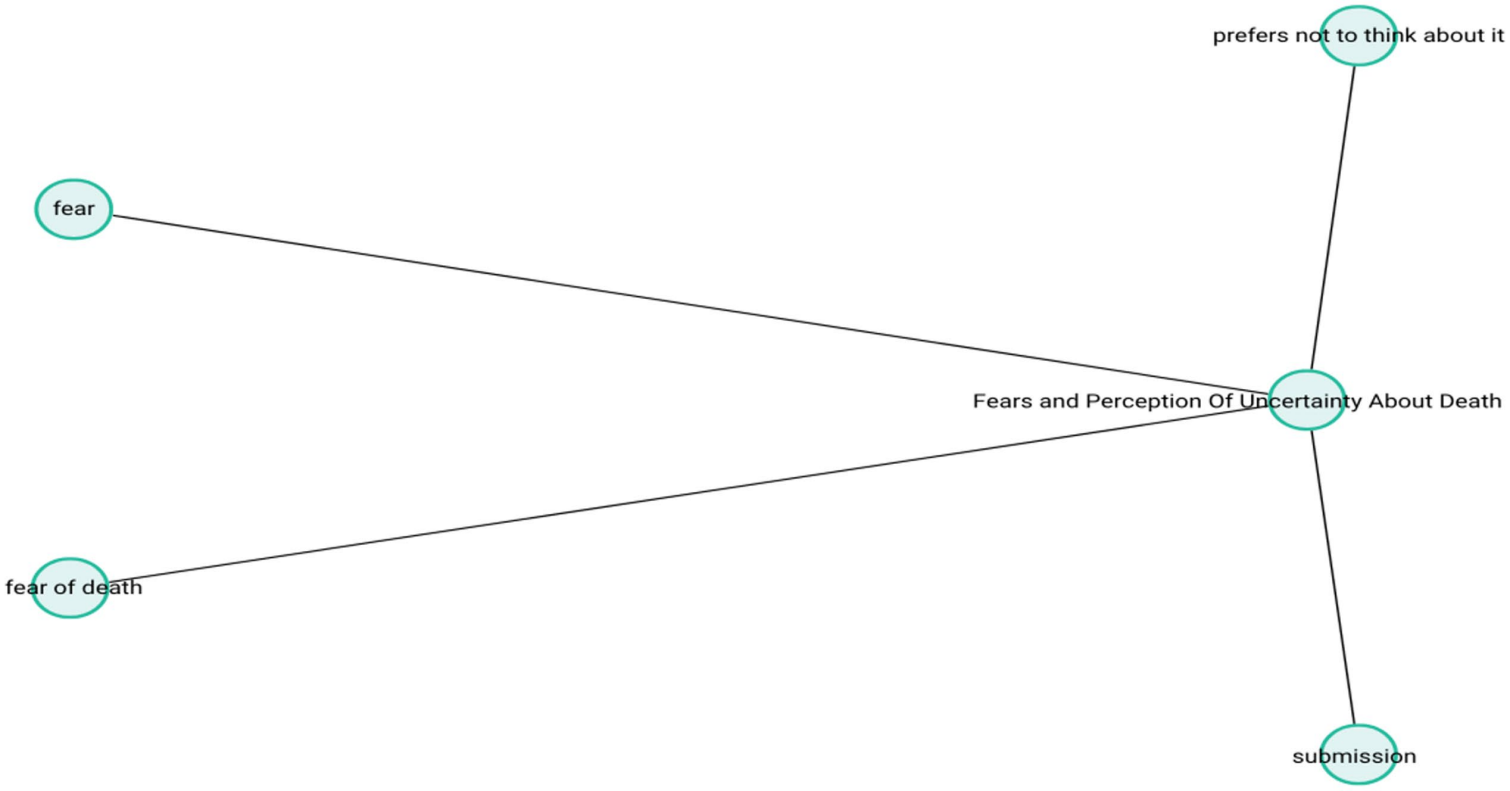
Fears and perception of uncertainty about death.

These narratives illustrate how death-related anxieties are often intertwined with fears of losing control, loneliness, unfulfilled life goals, and physical suffering. Among older adults, such concerns can manifest as a significant emotional burden impacting everyday well-being. The theme-subcode network (codemaps) of “Fears and Perception Of Uncertainty About Death” is presented in Figure 11.

### Theme 5: commitment to life and feeling of incompleteness

Finally, some participants expressed that thoughts of death evoked a sense of “unfinished business.” A total of 8 participants reflected on this theme. For these individuals, life was seen as an ongoing journey, or a task still awaiting completion:

P15/M: “*I think there are many things I have not experienced yet. Time has flown by like water.”*

P42/ F: “*The memories and loved ones I’ve left behind give meaning to my life*.”

P13/F: “*I want to live every moment to the fullest, surrounded by the people I love.”*

These responses underscore a longing to maintain a connection with the future and a concern that death might prematurely interrupt this continuity. The desire to stay alive is shaped by strong emotional ties to loved ones and the pursuit of life goals not yet fully realized.

**Figure 12:**
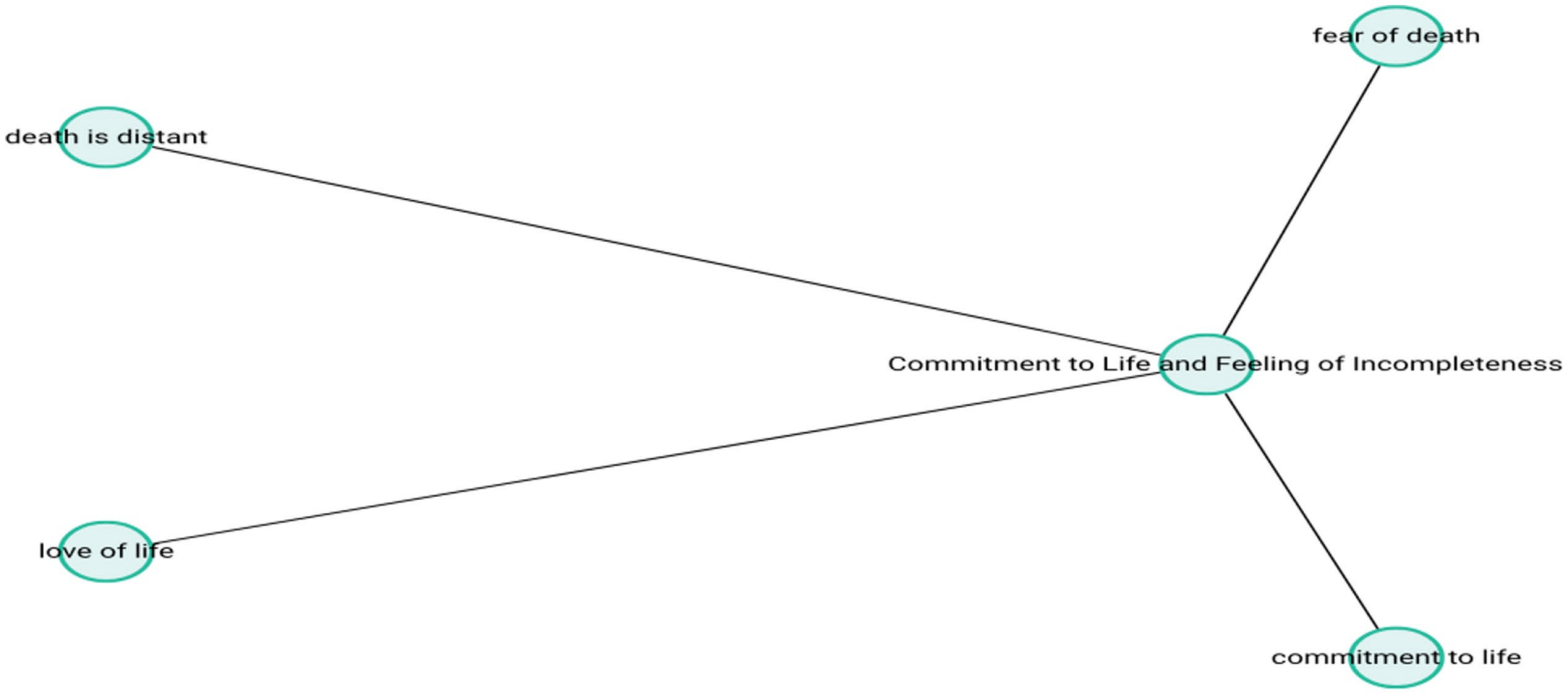
Commitment to life and feeling of incompleteness.

Collectively, these findings indicate that participants conceptualize death not merely as a physiological endpoint, but as a deeply layered experience shaped by religious belief systems, life satisfaction, physical health, and accumulated life experiences. For many, death was viewed not as something to be feared, but as an inevitable event for which one must prepare. However, a subset of participants still approached death with anxiety, uncertainty, and a sense of incompletion, reflecting the diverse and dynamic nature of late-life existential reflection. The theme-subcode network (codemaps) of “Commitment to Life and Feeling ofIncompleteness” is presented in Figure 12.

### Thematic patterns in relation to socio-demographic variables

When thematic findings are considered alongside key socio-demographic characteristics, it becomes evident that individuals’ meaning-making processes are closely intertwined with broader social and structural conditions. From a gender-based perspective, female participants predominantly clustered under the theme of Family and Intergenerational Meaning. Their narratives frequently emphasized motherhood, child-rearing, marital relationships, and familial bonds. In contrast, male participants were less represented within this theme, but expressed themselves more frequently under the theme of Occupational and Economic Independence, where they defined their identities in terms of professional achievement, financial autonomy, and social contribution.

Notably, regret narratives among women were strongly shaped by themes related to education and marital decisions. Many female participants described their most profound regrets as being unable to complete their education, being prevented from attending school, or being forced into early or unwanted marriages. These accounts reflect the lasting psychological impact of constrained decision-making autonomy across the life span.

Statements such as “I wish I had studied more,” “Getting married young changed my life,” and “It was my family’s decisionI wish I could have objected” reveal how the denial of educational opportunitiesand thus the potential for individualizationhas become one of the most persistent and deeply felt regrets among women. As such, these regret narratives function not only as reflections on personal choices but also as implicit critiques of gendered social constraints. The data suggest that participants raised in more traditional or rural environments became increasingly aware of these constraints in later life.

A preliminary analysis based on educational attainment revealed that participants with at least a high school education tended to express greater thematic diversity. They were more visible within themes such as Pride in Lifestyle Choices and Moral and Spiritual Fulfillment. In contrast, participants with lower educational backgrounds more frequently aligned with themes such as Family and Intergenerational Meaning, Relational and Familial Disruptions, and Faith-Based Acceptance. These findings suggest that educational level may be associated with more diverse and individualized forms of life evaluation. Regarding socio-economic status, participants who identified as “high-income” often emphasized financial success, long-term planning, and the ability to seize opportunities. Conversely, participants in the low-income group tended to express greater regret, missed opportunities, and emotional ruptures. Middle-income participants demonstrated a more balanced thematic distribution, reflecting both familial accomplishments and personal meaning-making.

In sum, the thematic structures that emerged from participants’ narratives reflect not only individual life experiences but also broader patterns shaped by social class, educational background, gender roles, and structural inequalities. These patterns underscore the importance of interpreting late-life reflections within their socio-cultural and socio-economic contexts.

## Discussion

### Question 1: life events that elicit pride (“I’m glad I did/experienced it”)

Participants’ reflections on life experiences they described as meaningful or pride-inducing provide valuable insights into how individuals construct meaning through retrospective self-evaluation. Their narratives reveal which experiences were most valued and how life satisfaction is articulated across different life domains. The analysis identified a set of interrelated dimensions encompassing family and intergenerational meaning, occupational and economic independence, autonomous lifestyle choices, and moral and spiritual fulfillment.

Family life—forming a family, having and raising children—emerged as the most frequently cited and deeply meaningful aspect of participants’ lives. This finding aligns with Erikson’s final psychosocial stage, *Ego Integrity versus Despair*, which highlights the importance of achieving coherence and meaning from one’s past, often rooted in familial continuity, social connectedness, and culturally valued roles (Erikson, 1982). Within the Turkish cultural context, where family constitutes a central institution and older adults are regarded with moral and cultural reverence, it is unsurprising that pride and fulfillment are often expressed through family-oriented narratives (Kagitcibasi, 2017). In collectivist cultures such as Turkey, ego integrity is shaped not solely by individual accomplishments but also by one’s contributions to family and society (İmamoğlu, 1998).

Equally significant was the theme of occupational and economic independence. Participants emphasized professional success, financial autonomy, and their contributions to family and community as key sources of meaning. Occupation was perceived not merely as a means of livelihood but as a foundation of personal identity and social responsibility. This reflects the continuity of Erikson’s concept of *generativity*—the task of being productive and contributing to others—into the stage of ego integrity (Erikson, 1982). Research suggests that occupational satisfaction fosters a sense of usefulness and meaning, thereby reinforcing life satisfaction (McAdams et al., 1997; Wrzesniewski and Dutton, 2001). Studies in Turkey further confirm that achievements during the generative years are reinterpreted in later life as expressions of personal worth, strengthening ego integrity (Kıyak, 2018; Mersin et al., 2018).

Another prominent dimension involved pride in lifestyle choices, including autonomy, simplicity, and alignment with personal values. Participants expressed contentment in having made independent decisions, maintained close friendships, and lived in accordance with their inner beliefs. These reflections support Erikson’s notion that ego integrity involves approving one’s life story and remaining faithful to one’s inner self (Erikson, 1982). Although Turkish society is largely collectivist, processes of urbanization and exposure to individualistic values have encouraged many older adults to interpret their autonomy and lifestyle choices as meaningful expressions of self (Göregenli, 1995; İmamoğlu, 2015).

Finally, moral and spiritual fulfillment played a crucial role in participants’ sense of meaning. Acting justly, helping others, caring for family members, and maintaining religious devotion were frequently cited as life-affirming behaviors. These expressions resonate with Erikson’s concept of *spiritual integrity*, which emphasizes spirituality’s protective function in fostering ego integrity during late adulthood (Hearn et al., 2012). The integration of moral and religious values into life narratives highlights the enduring significance of religiosity in Turkish experiences of aging. Prior studies indicate that religious belief serves as a psychological buffer against existential anxiety and supports ego integrity in later life (Orak et al., 2015; Yılmaz and Apalı, 2024). Moreover, caregiving—particularly devotion to aging parents—was often perceived as an act that enhances one’s sense of purpose and moral continuity (Hoeyberghs et al., 2019; Irving et al., 2017; Martos et al., 2010).

In sum, participants’ accounts demonstrate that meaningful life experiences are shaped by the intersection of familial, occupational, moral, and spiritual domains. These findings underscore the culturally embedded nature of ego integrity, revealing that for many older Turkish adults, life satisfaction arises not from isolated personal achievements but from an enduring sense of contribution, connection, and coherence across the lifespan.

### Question 2: life regrets (“I wish I had not done/experienced that”)

Participants’ reflections on regret, often articulated through phrases such as “I wish…,” provide a rich understanding of how individuals retrospectively evaluate their past decisions, missed opportunities, relational ruptures, and physical well-being over the life course. Their narratives reveal the psychological and cultural dimensions of regret and how unresolved reflections from the past may contribute to emotional distress or, conversely, serve as catalysts for self-acceptance and growth in later life.

A predominant source of regret involved missed opportunities in education, career, and personal development. Participants frequently expressed remorse over leaving school early, marrying at a young age, or failing to seize financial or professional opportunities. These reflections demonstrate a deep awareness of how past decisions have shaped present limitations, often accompanied by inner conflict and existential questioning. In Erikson’s framework, such realizations are intrinsic to the evaluative process of the *Ego Integrity versus Despair* stage; when unresolved, they may give rise to despair and self-doubt (Erikson, 1982). Research indicates that missed educational and occupational opportunities are strongly linked to perceived losses in social status, autonomy, and self-worth (Pinquart, 2002). Cross-cultural studies further support that regrets concerning education, marriage, and career are among the most common themes of remorse in later adulthood (Metha et al., 1989; Timmer et al., 2005). Within the Turkish context, these regrets are often amplified by structural and historical factors such as rapid urbanization, internal migration, and limited educational access—particularly for women—which collectively constrained life choices and intensified feelings of unrealized potential in older generations (Kavas and Thornton, 2013; Kurt et al., 2010; Mersin et al., 2018).

Another significant dimension of regret concerned relational and familial disruptions. Participants described disappointment in marriage, emotional disconnection from spouses or children, and unresolved family conflicts as enduring sources of distress. Such experiences align with Erikson’s conception of despair, particularly when emotional intimacy—a central developmental aim in later life—is undermined by relational discord (Erikson, 1982). In Turkish society, where family cohesion and interdependence are deeply valued, the deterioration of close relationships or persistent familial tension may profoundly exacerbate the experience of despair (Çevik Akyıl et al., 2018; Demir and Sağlık, 2022; Kagitcibasi, 2017). The emotional toll of restricted autonomy within traditional marital norms, including arranged marriages, further highlights how gendered and cultural expectations influence later-life regret and emotional well-being.

A third prominent source of regret centered on health and bodily neglect. Participants often expressed remorse for unhealthy habits such as smoking, alcohol consumption, or failure to attend medical check-ups. These accounts illustrate how earlier disregard for physical health becomes increasingly visible and emotionally burdensome with aging. Such reflections resonate strongly with Erikson’s notion of late-life retrospection—where individuals confront the consequences of past actions and grapple with the thought, “I wish I had done things differently” (Erikson, 1982). Extensive research confirms that unhealthy lifestyle choices during youth have enduring consequences for physical and psychological well-being (Nygren et al., 2005; Wurm et al., 2007). Turkish studies similarly reveal that older adults frequently attribute their present health problems to earlier neglect, expressing sentiments such as “I wish I had taken better care of myself,” which reflect both regret and recognition of personal responsibility (Karataş and Duyan, 2008; Küçük and Karadeniz, 2021).

Overall, participants’ narratives demonstrate that regret is not merely a reflection of individual missteps but a complex interplay of personal, relational, and structural factors. These findings underscore the culturally embedded nature of regret, showing that in the Turkish context, feelings of remorse often stem from constrained life circumstances rather than purely individual failings. Yet, through reflection and meaning-making, some participants also appeared to transform their regrets into acceptance, suggesting that regret, when integrated into the life story, may ultimately contribute to ego integrity rather than despair.

### Question 3: thoughts on death

Participants’ reflections on death provided profound insights into how mortality is conceptualized in late adulthood and how emotional, cognitive, and spiritual processes shape this understanding. Their narratives revealed diverse yet interconnected perspectives on faith, acceptance, fear, and the desire for completion. Collectively, these reflections illuminate how older adults navigate the final developmental challenge identified by Erikson—achieving ego integrity in the face of death (Erikson,1982).

For many participants, death was viewed through a spiritual lens, perceived not as an end but as a divine transition to another realm. They frequently emphasized faith-based acceptance and surrender, describing death as part of God’s will and a continuation rather than cessation of existence. This interpretation reflects a culturally and religiously grounded approach to mortality, consistent with Islamic views that frame death as predetermined (kader) and spiritually meaningful (Balcı, 2017; Gürsu and Ay, 2018). Such beliefs provide psychological comfort and stability, aligning with Erikson’s concept of ego integrity, in which individuals accept death as a natural and meaningful culmination of life. Empirical evidence confirms that religiosity functions as a protective factor against death anxiety, reinforcing a sense of purpose and coherence in old age (Jong, 2021; Thorson and Powell, 1990; Wink and Scott, 2005).

Participants also expressed specific wishes regarding how they would like to die, emphasizing the desire for a “good death” — one that is painless, dignified, and does not impose suffering on others. These reflections reveal a strong preference for maintaining personal autonomy and emotional wholeness at life’s end. Consistent with previous findings, older adults were often less fearful of death itself than of the dying process, especially concerns about pain, dependency, and loss of control (Carr, 2012; Fortner and Neimeyer, 1999). Within the Turkish context, the wish for a good death was closely tied to preserving dignity and avoiding dependency, highlighting the cultural value placed on self-sufficiency and family harmony (Altaş, 2020; Yılmaz and Mermutlu, 2023).

A significant number of participants described a state of peacefulness and readiness for death. They viewed it as a natural and inevitable conclusion to a completed life cycle. This acceptance, which Erikson considered the hallmark of ego integrity, reflects emotional maturity and reconciliation with one’s life story (McLeod-Sordjan, 2014; Parker, 2013). Older adults who had achieved a sense of coherence and meaning expressed less fear of death and a greater willingness to prepare for it consciously (Abreu et al., 2022; Krok and Brudek, 2023). Turkish studies similarly indicate that individuals with higher ego integrity report greater life satisfaction and a deeper sense of peace toward mortality (Akbolat, 2021; Yılmaz and Mermutlu, 2023).

Nevertheless, some participants expressed fear, uncertainty, and avoidance regarding death. Concerns about suffering, loss of control, or becoming a burden to loved ones were prominent. Such responses align with Erikson’s notion of despair, arising when individuals struggle to find meaning in their lives or perceive unfulfilled moral and relational duties (Erikson, 1982; Fishman, 1992). Empirical studies have shown that lower levels of ego integrity correspond with heightened death anxiety and reduced psychological well-being (Sekowski, 2022). Within the Turkish-Islamic framework, however, belief in divine destiny and the afterlife serves as a crucial coping resource, allowing individuals to contextualize mortality within a spiritual narrative and mitigate existential fear (Fischer and Secinti, 2022).

Finally, a recurring theme was the attachment to life and a sense of incompletion. Some participants expressed reluctance to die, citing strong emotional ties to loved ones or a feeling that certain goals remained unfulfilled. This tension often represented an unresolved internal conflict and corresponded with Erikson’s concept of despair. Research suggests that perceiving one’s life as incomplete intensifies fear of death and existential anxiety (DeGenova, 1992; Zhang et al., 2019). Turkish studies further reveal that older adults who feel they have unfinished responsibilities or unattained aspirations experience greater distress when contemplating mortality (Akın, 2018; Arpacı et al., 2011).

Taken together, these narratives illustrate the multifaceted nature of how older adults in the Turkish context engage with mortality. Faith, acceptance, and moral reflection serve as mechanisms for achieving peace and ego integrity, whereas unresolved regret, dependency fears, or a sense of incompletion may foster despair. Ultimately, the participants’ reflections demonstrate that attitudes toward death are deeply embedded within cultural, spiritual, and psychosocial frameworks that shape how individuals seek meaning and coherence at life’s end.

## Conclusion

Grounded in Erikson’s psychosocial theory of development, this study examined how older adults in the Turkish cultural context evaluate their life experiences, articulate sources of pride and regret, and conceptualize death. The findings demonstrate that the process of achieving ego integrity in later life is not solely an individual endeavor but one that is deeply embedded in sociocultural, economic, and religious frameworks.

Participants’ sense of pride was primarily anchored in family accomplishments, professional productivity, and adherence to moral and spiritual values. Regrets, by contrast, most frequently stemmed from limited access to education, relational disruptions, and neglect of physical health. Reflections on death revealed that faith-based acceptance often facilitated a sense of peace and readiness, whereas some participants continued to experience existential anxiety and feelings of incompletion. Collectively, these themes correspond closely with Erikson’s dialectical constructs of ego integrity and despair, highlighting the dual potential for reconciliation and distress in late adulthood.

The results underscore the significance of integrating faith-sensitive counseling and spiritually informed psychoeducational approaches within psychosocial support systems for older adults. Such interventions could promote coherence in self-narratives, enhance life satisfaction, and foster a more tranquil acceptance of mortality. In addition, socio-demographic factors including gender, education, and socioeconomic status were found to influence the thematic expression of meaning and regret. Female participants more often reflected on family and educational constraints, whereas male participants tended to associate ego integrity with professional achievement and social contribution. Higher levels of education and economic stability were associated with greater depth and diversity in life reflections, suggesting that social context profoundly shapes late-life meaning-making.

These findings further point to the importance of family-centered intervention strategies aimed at strengthening intergenerational communication, repairing emotional ruptures, and fostering reciprocal understanding among aging family members. In particular, self-reflective psychosocial support groups for older women, emphasizing self-efficacy and guided life review, may provide meaningful opportunities for psychological integration and empowerment. In aging societies such as Turkey, the development of culturally responsive social policies and gerontological care models that nurture meaningful late-life experiences is essential. Incorporating a psychosocial integrity framework into national aging policies could help ensure that older adults are supported not only in their physical and economic needs but also in their quest for meaning, coherence, and spiritual peace in the final stages of life.

### Strengths and limitations

This study provides a valuable contribution to the field of aging psychology by integrating Erikson’s theory of ego integrity and despair within a non-Western, collectivist cultural context—an area that remains underrepresented in gerontological research. It offers one of the few qualitative examinations of how older adults in Turkey construct meaning, reconcile life experiences, and reflect on mortality within their sociocultural and spiritual frameworks. The use of qualitative narrative analysis is a notable strength, as it captures the subjective, emotional, and moral dimensions of aging that quantitative measures often overlook. By analyzing written life narratives, the study gives voice to participants’ lived experiences, allowing for a nuanced understanding of ego integrity as a culturally embedded and dynamic process. Moreover, the study’s contextual sensitivity—its attention to religiosity, interdependence, and moral responsibility—advances the cross-cultural applicability of Erikson’s framework. The findings have direct implications for designing faith-sensitive psychosocial interventions and gerontological support programs that align with the values and belief systems of older adults in collectivist societies.

Despite these strengths, several limitations should be noted. The study’s sample size was relatively small and limited to urban-dwelling older adults, which may constrain the generalizability of findings to rural or socioeconomically diverse populations. As with most qualitative, cross-sectional research, the results are interpretive and context-specific, reflecting participants’ retrospective perceptions rather than longitudinal developmental trajectories. Researcher subjectivity and narrative selectivity may have influenced theme construction despite efforts toward reflexivity and analytic rigor. Furthermore, the study did not incorporate health-related variables (e.g., cognitive or physical functioning), which could interact with psychosocial well-being in late life. Future research integrating mixed methods, larger and more diverse samples, and longitudinal designs would strengthen causal inference and enhance understanding of ego integrity across different cultural and developmental contexts.

## Data Availability

The raw data supporting the conclusions of this article will be made available by the authors, without undue reservation.
